# Antidiabetic Drug Sitagliptin with Divalent Transition Metals Manganese and Cobalt: Synthesis, Structure, Characterization Antibacterial and Antioxidative Effects in Liver Tissues

**DOI:** 10.3390/cimb44050124

**Published:** 2022-04-20

**Authors:** Samy M. El-Megharbel, Najah M. Al-Baqami, Eman H. Al-Thubaiti, Safa H. Qahl, Bander Albogami, Reham Z. Hamza

**Affiliations:** 1 Department of Chemistry, College of Sciences, Taif University, P.O. Box 11099, Taif 21944, Saudi Arabia; 2 Department of Biological Sciences, Zoology, Faculty of Sciences, King Abdul-Aziz University, P.O. Box 80200, Jeddah 21589, Saudi Arabia; nalbogami@kau.edu.sa; 3 Biotechnology Department, College of Sciences, Taif University, P.O. Box 11099, Taif 21944, Saudi Arabia; i.althubaiti@tu.edu.sa; 4 Biology Department, College of Sciences, Jeddah University, P.O. Box 34, Jeddah 21959, Saudi Arabia; shqahal@uj.edu.sa; 5 Department of Biology, College of Sciences, Taif University, P.O. Box 11099, Taif 21944, Saudi Arabia; b.boqami@tu.edu.sa (B.A.); reham.z@tu.edu.sa (R.Z.H.)

**Keywords:** diabetes mellitus, sitagliptin, metal complexes, glycemic state, HbA1C

## Abstract

Metals and their complexes have an increasing number of medical applications. Sitagliptin (STG) acts as an antidiabetic drug. Mn(II) and Co(II) complexes were studied and characterized based on physical characterization, FT-IR, DG/TG, XRD, ESM, and TEM. Data revealed that STG acts as a bidentate ligand through the oxygen atom of a carbonyl group and the nitrogen atom of an amino group. Magnetic measurement data revealed that the Mn/STG metal complex has a square planner geometry. The experiment was performed on 40 male albino rats who were divided into four groups: the control group, STG group, group treated with STG/Mn, and group treated with Co/STG. Biomarkers for hepatic enzymes and antioxidants were found in the blood, and hepatic tissue histology was evaluated. STG in combination with Mn and Co administration showed potent protective effects against hepatic biochemical alterations induced by STG alone, as well as suppressing oxidative stress and structural alterations. These complexes prevented any stress and improved hepatic enzymatic levels more than STG alone. The STG/Mn complex was highly effective against *Bacillus subtilis* and *Streptococcus pneumonia*, while STG/Co was highly effective against *Escherichia coli*, *Pseudomonas aeruginosa,* and *Staphylococcus aureas*. Therefore, STG combined with Mn and Co produced a synergistic effect against oxidative stress and improved the histological structure of the liver tissues. STG metal complexes with Mn and Co showed the most potential ameliorative antioxidant and hepatoprotective effects.

## 1. Introduction

Sitagliptin (STG) was the first dipeptedyl peptidase-4 inhibitor approved by the FDA for the management and treatment of type II diabetes mellitus [1]. It is broadly used as an adjuvant therapy to current treatment [2]. STG has been shown to inhibit the expression of inflammatory mediators and suppress NF-κB activation. Previous Japanese reports and some international reports (MEDLINE) have linked STG treatment to an increase in hepatic transaminases [3].

STG is the first gliptin-based agent approved in the USA in 2006 and Europe in 2009. Additionally, STG has the advantage of being an anti-inflammatory agent [4,5].

STG was developed as a new type II diabetes mellitus therapeutic option. STG is a type of antidiabetic agent that was only recently approved for diabetes treatment [6]. In addition to regulating incretins, STG has various functions in different organs [6].

Dipeptedyl Peptidase-4 is also involved in the development of chronic hepatitis-C (HIV) [7,8]. Additionally, serum dipeptedyl peptidase-4 activity was found to be increased in tetrachloromethane-induced cirrhotic rats [9]. Dipeptedyl peptidase-4 categories are highly expressed in the liver and are involved in the fibronectin-mediated interaction of hepatocytes with ECM [10,11]. However, the effect of STG on the advancement of liver fibrosis is still unknown. The hepatic tissues highly express dipeptedyl peptidase-4, and recently dipeptedyl peptidase-4 drugs were suggested to be involved in the development of many chronic hepatic diseases, carcinoma, and even fatty liver [10].

The peptidase activity of the enzyme dipeptidyl peptidase-4 exerts pleiotropic effects on many organs. Recent data have suggested that STG plays a role in the development of fatty liver diseases, chronic liver diseases, and hepatocellular carcinoma [11]. STG is a relatively new generation of antidiabetic drug that inhibits dipeptidyl peptidase-4 enzymatic activity, resulting in continuous rapid degradation of GLP-1 [12].

The high expression of dipeptidyl peptidase-4 in many tissues, including lymphocytes, has raised the effectiveness of STG, particularly on immunological functions [13]. As is well known, STG is used to treat patients with diabetes mellitus, and the nature of the disorder itself increases the risk of many organ-related complications, confirming the observed hepatic structural alterations induced after STG treatments.

Inhibition of dipeptidyl peptidase-4 by gliptins prolongs endogenous glucose-dependent insulinotropic polypeptide (GIP) activity, lowering blood glucose levels in diabetic patients [14].

Patients with type II diabetes mellitus have a twofold increased chance of developing hepatic fibrosis or hepatocellular carcinoma compared to the general population. Additionally, the risk of dying from hepatic fibrosis is doubled in patients with type II diabetes mellitus [15].

Hepatic STG expression in the liver may be directly associated with hepatic lipogenesis and liver injury. STG, an oral antihyperglycemic agent, inactivates the hormones glucagon-like peptide-1 (GLP-1) and GIP released in response to meals. STG increases insulin secretion and suppresses glucagon, which lowers blood glucose levels by preventing GLP-1 and GIP breakdown [16].

A previous study demonstrated that novel synthesized Mg, Cr, Cu, Ca, Zn, and Se/STG complexes significantly improved insulin secretion and the pancreatic and glycometabolic functions and succeeded in controlling high fat diet-induced diabetes mellitus in rats. It showed that there were great differences in the biochemical and histological findings obtained from the diabetic rats orally receiving STG and those receiving STG–metal complexes, and there was improved insulin secretion of diabetic rats than in the ones given STG only and enhanced antioxidant capacities after the induction of experimental diabetes mellitus in male rats and alleviated complications of diabetic rats than STG alone [5].

Carbon, hydrogen, and nitrogen elemental analysis; infrared spectroscopy, molar conductance measurement, and magnetic moment were used to investigate STG complexity. The spectroscopic results suggested octahedral structures for prepared Ni–STG complexity. The chemical structure of STG complexity was elucidated by Fourier-transform infrared spectroscopy (FT-IR), X-ray diffraction (XRD), scanning electron microscopy (SEM), and transmission electron microscopy (TEM). This study is aimed at synthesizing Mn(II) and Co(II) STG complexes and testing their hepatoprotective and antioxidant effects besides antibacterial activity of the two synthesized complexes.

## 2. Materials and Methods

### 2.1. Chemicals

Chemicals that were obtained from commercial suppliers (Sigma-Aldrich (St. Louis, MO, USA) Fluka (Buchs, Switzerland) or Merck Chemical Company (Buchs, Switzerland)) and used as supplied were used without further purification. STG (C_16_H_15_F_6_N_5_O; 407.31 g/mol) [purity 98%; Metals(II) chlorides] was obtained from Fluka (purity 98%).

### 2.2. Preparation of Divalent Metal STG Complexity

Each STG ligand (0.02 mol, 8.15 g) dissolved in 25 mL CH_3_OH was combined with divalent Mn^+2^ (0.01 mol, 1.26 g) for first solution mixture and Co^+2^ (0.01 mol, 1.30 g) dissolved in 50 mL CH_3_OH for second solution mixture and then stirred with refluxing for 2 h for each mixture. The produced precipitates were separated by evaporating the precipitated solution to 1/2 of its original volume (v), resulting in a brownish-white precipitate for Mn^+2^ and a pale-yellow precipitate for Co^+2^. For the complexes, filtration, multiple washing with MeOH solvent, and drying at 60 °C with a vacuum desiccator over anhydrous CaCl_2_ were carried out and chemically characterized by different instruments as shown in Table 1.

#### 2.2.1. Compound 1 (Mn^+2^ Complex)

Yield (70%), m.p. > 300 °C; color: brownish; molar conductivity (Λm(μS)): 68 ohm^−1^ cm^2^ mol^−1^. Elemental analysis for [Mn(STG)_2_]. Cl_2_, MnC_32_H_30_N_10_Cl_2_F_12_O_2_, (940.48 g/mol): calcd. (Found)%C 40.87 (40.22), %H 3.22 (3.01), %N 14.89 (14.53), %Cl 7.54 (7.53), and %Mn 5.84 (6.68); IR values in cm^−1^ using KBr tablets: 3330 ν (NH), 1637 ν (C=O), 1633 ν (C=N)imine, 1512 ν (NH), 1333, 1269, 1140 ν (C–F), 660 ν (Mn–O), 560 ν (Mn–N), UV–Vis. (1 cm quartz cells, DMSO) 203, 260 nm.

#### 2.2.2. Compound 2 (Co^+2^ Complex)

Yield (70%), m.p. > 300 °C; color: pale-yellow; molar conductivity (Λm (μS)): 67 ohm^−1^ cm^2^ mol^−1^. Elemental analysis for [Co(STG)_2_]. Cl_2_, CoC_32_H_30_Cl_2_F_12_N_10_O_2_, (944.47 g/mol): calcd. (Found)%C 40.69 (40.32), %H 3.20 (3.05), %N 14.83 (14.63), %Cl 7.51 (7.63), and %Co 6.24 (6.78). IR values in cm^−1^ using KBr tablets: 3331 ν (NH), 1638 ν (C=O), 1633 ν (C=N) imine, 1510 ν (NH), 1332, 1270, 1139 ν (C–F), 661 ν (Co–O), 558 ν (Co–N), UV–Vis. (1 cm quartz cells, DMSO) 202, 261 nm.

The melting point of complexes was measure via A melting point apparatus which is a scientific instrument used to determine the melting point of complexes, Fisher-Johns apparatus, Gallenkamp (electronic) melting point apparatus, and automatic melting point apparatus.

### 2.3. In Vitro Release Profile of STG/Mn and STG/Co Complexes

The release profile of STG/Mn and STG/Co complexes were studied by using two compartment diffusion cells separated by a cellulose membrane of molecular weight cut-off = 12.000. A total of 3 mg of the prepared complexes were suspended in the donor compartment in 1 mL slightly basic PBS, whereas the receiver compartment contained PBS and was left shaking at 37 °C for about 8 h. The amount of both complexes released from the dialysis bag was determined at different intervals by measuring the absorbance values at 390 nm. All the measurements were done in triplicates [17].

### 2.4. Sample Handling for Flame Atomic Absorption Spectrometry (FAAS)

Preparation of the sample is the most important step in the analysis and involves simple dilution to total dissolution including wet decomposition of the solid sample using thermal, ultrasonic, or radiant infrared energy, where the brand/model for flame atomic absorption instrument is Perkin Elmer Analyst 400. A typical sample preparation procedure for solid and viscous liquid samples involves digestion with a concentrated acid, for example, HNO_3_, HCl, or H_2_SO_4_. After dilution of the digested solutions, samples can be directly injected into flame AAS as well as graphite furnace AAS.

For solid samples, many steps are required, including sampling, subsampling, grinding, and dissolution. The risk of contamination is higher than in the case of liquid samples. Sample preparation can be performed with parts per million (ppm) as concentration unit; it is necessary to add reagents to the solid sample and apply enough energy to break some present in solids. It is possible to use complementary reagents to prepare solid sample in solution using wet decomposition [18,19].

#### Wet Dissolution

Oxidizing agents were used to decompose solid organic samples. Concentrated acids such as HNO_3_, HCl, H_2_SO_4,_ and H_3_PO_4_ are used with one normal and make heating where the acid acts as complexing and oxidizing power, its boiling point safety and purity. The boiling point of the acid for open vessel decomposition controls the maximum temperature to be used to overcome high acid consumption and possible losses of the volatile contents [18,19]. Perchloric acid, if used, should never be more concentrated than 72% in the presence of organic matter due to the risk of explosion. The experiment can be performed using different types of energy such as thermal, ultrasonic, and radiant energy. Liquid samples are aspirated and introduced into the flame using a spray chamber that makes breaking for the aspirated liquid and changes it into fine droplets.

### 2.5. Experimental Model

**Figure 1:**
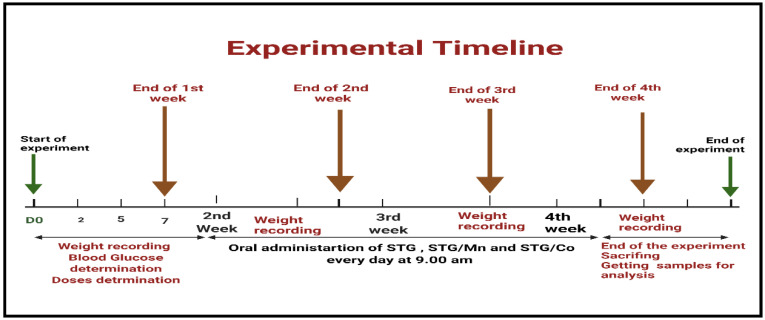
Timeline for experimental protocol.

Forty adult male albino rats weighing 140 g were maintained in metal cages in a pathogen-free environment with adequate ventilation. The animals were fed a healthy diet. During the experiments, every effort was taken to make the animals as comfortable as possible. The experimental protocol was approved by the Taif University Ethical Committee (approval number: 42-0074) and followed international guidelines on animal use and care. The animals were provided with food and water *ad libitum* (Figure 1).

### 2.6. Drugs and Chemicals

STG in the form of JANUVIA^®^ 100 tablets was obtained from Merck Sharp and Dohme Ltd. (Pavia, Italy). Each tablet was ground and dissolved in 10 mL carboxymethyl cellulose solution (0.5%) and subsequently shaken to achieve a suspension form (10 mg/mL).

### 2.7. Experimental Design

**Figure 2:**
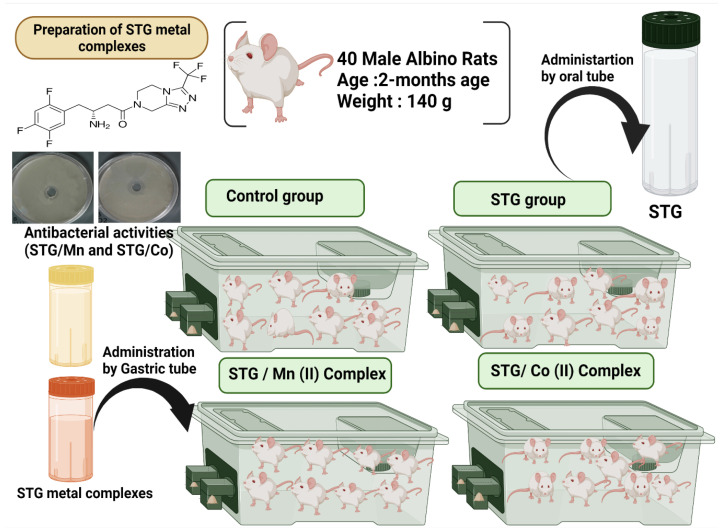
Graphical abstract for the experimental design.

The animals (2 months old) were divided into four treatment groups. All treatments were administered orally for 30 successive days (4 weeks). Control group (I) received normal physiological saline solution (0.9%) with gum acacia as a vehicle; group (II) received STG [Sigma-Aldrich, St. Louis, MO, USA (10 mg kg^−1^)] [5]; group (III) received STG/Mn(II) (10 mg/kg); and group (IV) received STG/Co(II) at recommended doses, all treatment combined with gum acacia (carrier Vehicle). The treatment protocol is shown in Figure 2.

#### 2.7.1. Blood Samples

After the end of the experiment, blood samples were taken from the rat eye plexus and separated into two parts. One part was added to tubes with EDTA to obtain plasma, and the other part was added to EDTA-free tubes centrifuged at 5000× *g* rpm for 15 min to obtain serum. Both samples were used for different biochemical analyses.

#### 2.7.2. Hepatic Function Activities and Biomarkers

ALT, AST, and ALP levels were assessed by using (SENTINEL CH) kits. LDH levels were measured in accordance with the manufacturer’s instructions.

#### 2.7.3. Preparation of Hepatic Tissue Homogenates for the Determination of the Redox State

Small liver portion from hepatic tissues were used to determine oxidative injury. The hepatic tissues were immersed in a 50 mM phosphate buffer (pH = 7.4), added with protease inhibitor to protect the enzymes from oxidation, and centrifuged to obtain the supernatant of tissue homogenates of brain and testicular tissues.

#### 2.7.4. Determination of Oxidative Stress Biomarker Activities in Hepatic Tissues

MDA level was determined following the method of Ohkawa et al. (1979) [20]. SOD activity was determined using the technique of Marklund and Marklund (1974) [21]. CAT activity was estimated by applying the method of Aebi (1984) [22]. GRx was determined following Couri and Abdel-Rahman (1980) [23]. Glutathione peroxidase (GPx) was assayed using the technique of Hafeman et al. (1974) [24].

#### 2.7.5. Histological Changes

Small hepatic tissues were fixed in 10% buffered formalin for further histological examination [25].

### 2.8. Antibacterial Activities of STG and Its Metal Complexes

The antimicrobial activity of the tested samples was determined by a modification of the Kirby–Bauer disc diffusion method [26]. Antibacterial activity was tested in triplicate, and then the mean was calculated. In brief, 100 μL of the best bacteria were grown in 10 mL of fresh media until they reached a count of approximately 108 cells/mL [27]. Then, 100 μL of the microbial suspension was spread onto agar plates corresponding to the broth in which they were maintained. Isolated colonies of each organism that may play a pathogenic role were selected from the primary agar plates and tested for susceptibility by the disc diffusion method [28].

Plates were inoculated at 25 °C for 48 h. The Gram-positive bacterium *Bacillus subtilis* and the Gram-negative bacterium *Escherichia Coli* were incubated at 35–37 °C for 24–48 h. Afterward, the diameters of the inhabitation zones were measured in millimeters [29]. Standard discs of tetracycline (antibacterial agent) served as positive controls for the antimicrobial activity, and a filter disc impregnated with 10 μL solvent (dist. H_2_O, chloroform, and DMSO) was used as a negative control.

The agar used was the Mueller–Hinton agar, which was tested continuously in terms of its pH. Further, the depth of the agar in the plate was a factor considered in the disc diffusion method [29].

### 2.9. Statistical Analysis

Statistical analysis was done by using SPSS software [30] and Open Epi version 2.3.1. One-way ANOVA and post hoc power were used to analyze data. *p* < 0.05 value was considered as significant [30].

## 3. Results and Discussion

### 3.1. Structural Characterization of the STG Metal Complexes

#### 3.1.1. Physical and Microanalytical Data

At room temperature, STG complexity had good stability and poor solubility in H_2_O solvent, but high solubility in organic solvents, such as DMF and DMSO. The molar conductance of the compounds 1 and 2 is 68 and 67 μs/cm, respectively. Based on electrolytic measurements of the compounds 1 and 2, Cl^−^ anions exist outside the chelation sphere and have a coordinated nature [31]. Adding AgNO_3_ reagent to the dissolved STG metal complexity produced a white precipitate, confirming the presence of Cl^−^ in the outer coordination sphere. The elemental analysis revealed a metal to ligand ratio of 1:2 in the divalent STG complexity.

#### 3.1.2. Infrared (IR) Spectroscopy of STG

**Figure 3:**
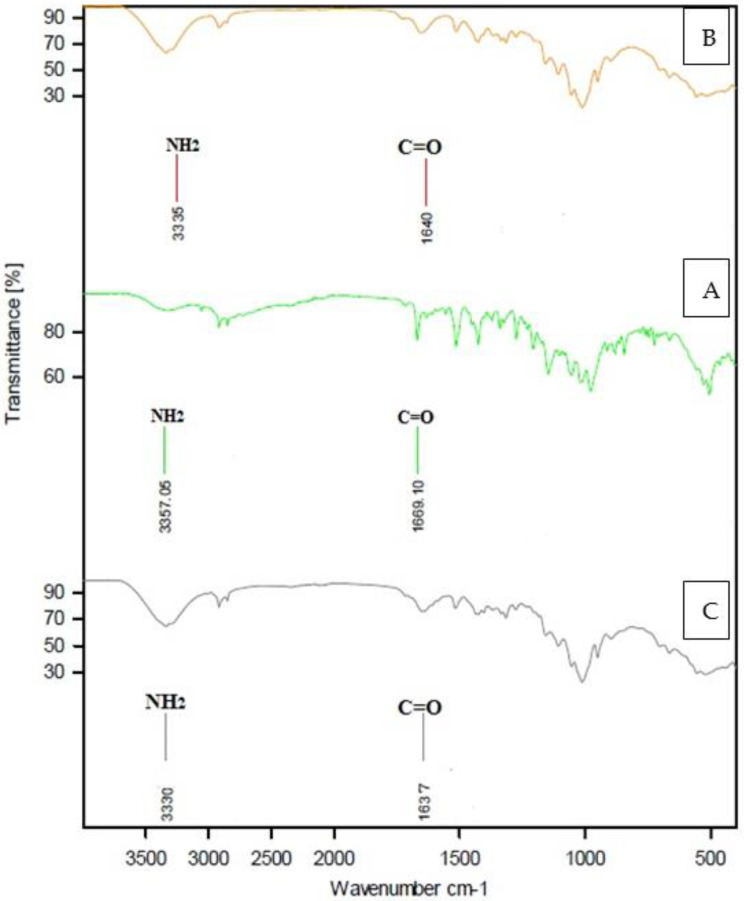
FT-IR of (**A**), STG; (**B**), STG/Co; and (**C**), STG/Mn.

The following were observed in the IR spectrum of the STG-free ligand (Table 2 and Figure 3): A strong spectrum with a broad character returned to the NH_2_ group (N–H stretching) at 3357 cm^−1^ [32], medium-strong and weak bands at 3050–3000 cm^−1^ due to aromatic C–H stretching and υ (C–H) vibrations, a medium-strong band at 1669 cm^−1^ corresponding to υ (C=O) stretching, a strong band at 1580 cm^−1^ returned to N–H bending, and one band at 1633 cm^−1^, representing the υ (C=N) azomethine group. The vibrations at 1000–1275 cm^−1^ were due to the υ (C-F) bond [33]. Other bands appeared at 1555, 1513, 1452, 1272, 1146, 978, 880, and 844 cm^−1^.

#### 3.1.3. IR Spectroscopy of STG Complexity

The following were observed in the IR spectra of STG metal complexity (Table 1 and Figure 3).

For compounds 1 and 2, the stretching vibration of the –NH_2_ group was displaced to lower frequency values at 3330 cm^−1^, 3331 cm^−1^, and 3335 cm^−1^, returning to chelation. In compounds 1 and 2 spectra, the carbonyl group [υ (C=O)] stretching at 1669 cm^−1^ (STG-free ligand) was shifted to a lower vibration at 1637–1640 cm^−1^ [33]. For the STG ligand, the band at 1633 cm^−1^ corresponds to the azomethine group (C=N), which is unshifted for compounds 1 and 2; therefore the azomethine group does not contribute to coordination. At 1330–1137 cm^−1^, the free STG revealed a band in the C–F region. After complexation, the maximum remained unchanged, indicating that the C–F bond was unlikely to be involved in complexity [33]. FTIR spectra showed small N–H region IR bands that cannot be attributed clearly to the coordination change, but easier to the H-bonding as well as the formation characteristic stretching vibrations due to N–H and C=O bonds.

**Figure 4:**
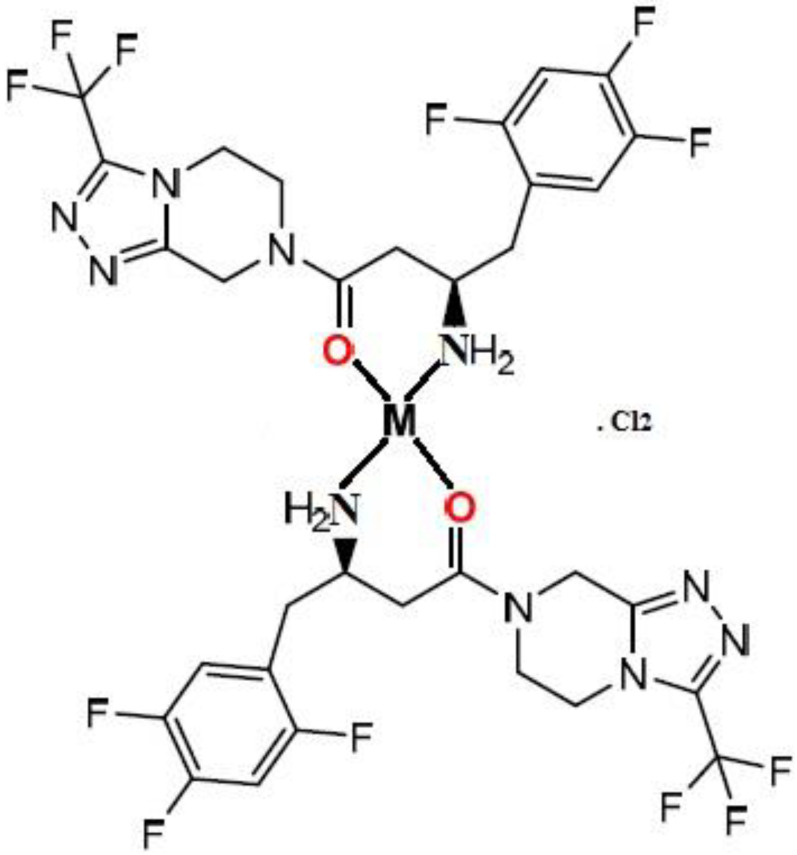
The proposed structure of complexes. where M = Mn^+2^ and Co^+2^.

The bands that emerged in the region of 666–655 cm^−1^ and at 548–558 cm^−1^ can be attributed to M–O and M–N stretching vibrations [34]. As a result of the IR spectral results, it was determined that the divalent metal ions (Mn^+2^ and Co^+2^) are coordinated with STG via amino and carbonyl groups, and STG functions as a bidentate chelate in trans form rather than cis (Figure 4) as trans structure is more stable than cis; in cis conformation, the bulky similar rings are on the same side that make steric repulsion of the rings that make cis conformation less stable than trans in which the bulky rings are far apart (on the opposite side).

### 3.2. XRD, SEM, and TEM Investigations

**Figure 5:**
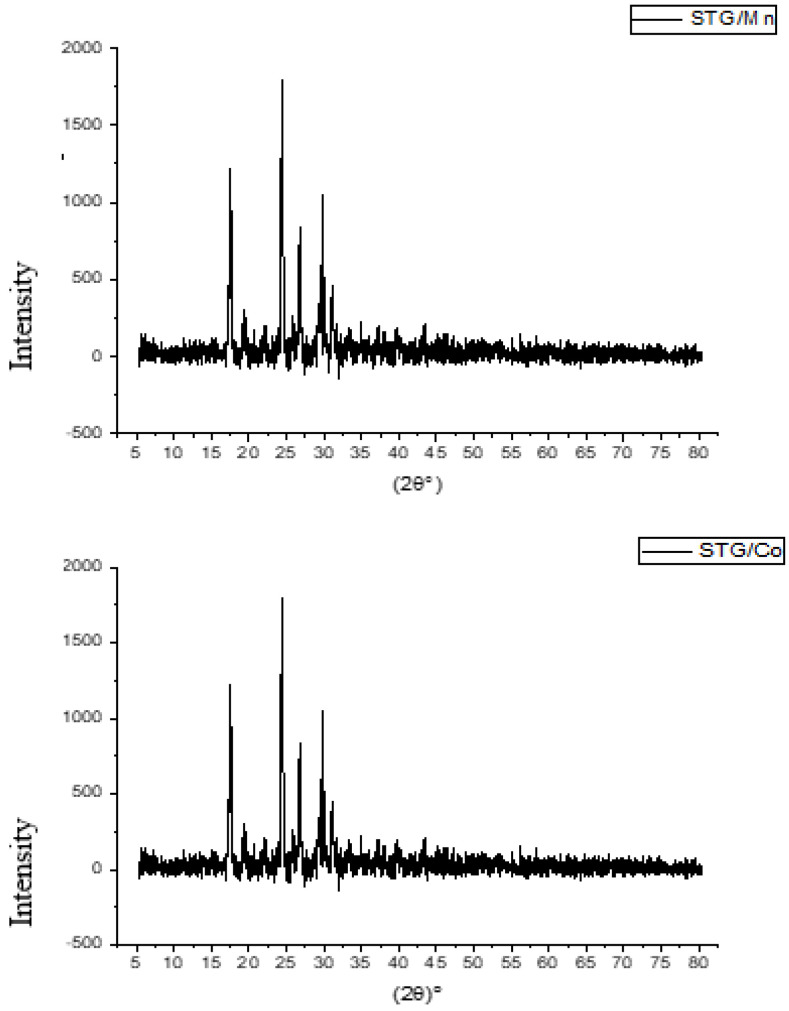
XRD of STG/Mn and STG/Co.

The samples of STG solid complexes were characterized at room temperature by X-ray diffraction by using Cu Kα radiation. A crystalline form of STG a powder X-ray diffraction pattern showed peaks at 2-theta of 13.7, 18.0, 22.6, 25.7, and 27.0 degrees. The nature of crystallinity for metal complexity was established by XRD, SEM, and TEM analyses. At room temperature, the compounds 1 and 2 were characterized by XRD using Cu Kα radiation. The XRD patterns of the synthesized STG complexity were crystalline. The diffractograms of compounds 1 and 2 have distinct patterns at (21.53, 24.21, 27.75, 28.65, 29.84, 31.32, 34.81, 38.92, 43.11, and 58.89 degrees) and (21.23, 24.93, 28.15, 29.45, 30.51, 32.61, 36.431, 38.32, 41.91, and 59.32 degrees) (Figure 5). A crystalline form of STG-free ligand showed. By making a comparison for compounds 1 and 2 with the X-ray patterns of Mg^2+^, Ca^2+^, Cr^3+^, Zn^2+^, Cu^2+^, and Se^4+^,STG complexes have distinct patterns at (14.94, 22.75, 25.35, 26.59, 30.27, 32.62, 40.22, 49.40, and 53.10 degrees), (22.75, 25.35, 26.61, 28.59, 30.28, 32.55, 32.96, 36.11, 40.15, 41.08, 47.42, 49.27, and 53.13), (22.43, 23.11, 25.45, 26.66, 28.67, 30.30, 32.87, 36.22, 40.17, and 53.09 degrees), (15.21, 22.75, 26.58, 28.51, 30.21, 32.75, 40.22, 49.27, and 52.96 degrees), (16.65, 21.11, 22.06, 22.54, 25.22, 26.46, 28.64, 28.64, 30.11, and 32.68 degrees), and (16.24, 19.39, 20.42, 22.54, 25.56, 26.63, 30.27, 32.68, 36.18, and 53.10 degrees), respectively. We elucidated the iso-crystalline structure for compounds 1 and 2, which could help elucidate the coordination geometry of the compounds.

The diffractograms of compounds 1 and 2 are depicted. The presence of sharp, intense, and strong Bragg’s diffraction lines in the diffractograms of STG complexity was well-characterized in nature and had a well-organized structure.

The crystalline size of the synthesized complexity was calculated using the Scherrer formula [29] D = kλ/βCosθ, where k is a constant of 0.94, λ is the wavelength of the X-ray used (0.154 nm), and β is the complete width of the XRD pattern at half maximum peak. Manganese and cobalt had crystalline sizes of 40 nm and 65 nm, respectively. The crystalline size was observed to be different for this complexity owing to changes in the metal ions.

**Figure 6:**
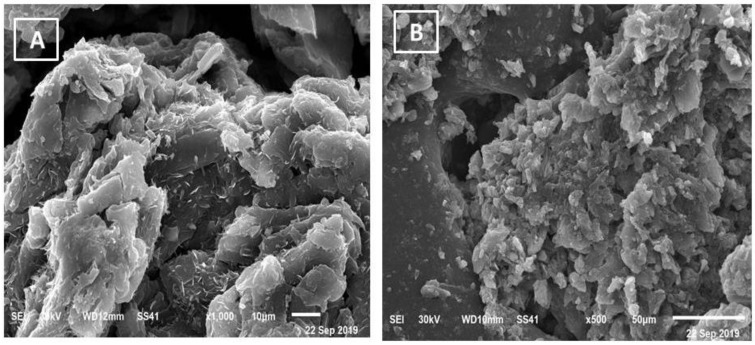
SEM of (**A**) STG/Mn and (**B**) STG/Co.

**Figure 7:**
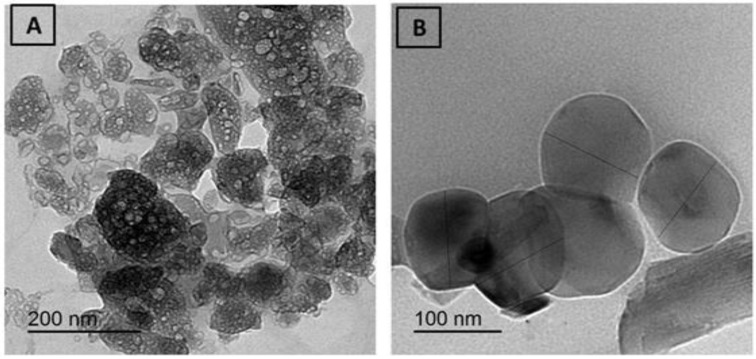
TEM of (**A**) STG/Mn and (**B**) STG/Co.

The SEM and TEM images of the compounds 1 and 2 are shown in Figure 6 and Figure 7. The average grain length for the compounds 1 and 2 is 5–50 μm, as can be seen in this diagram. The surface morphology changed as metal ions varied, with images having a large number of irregularly shaped grains and others having uniform grains. The average grain size estimated by SEM is quite a bit larger than the average grain size estimated by XRD.

### 3.3. In Vitro Drug Release

**Figure 8:**
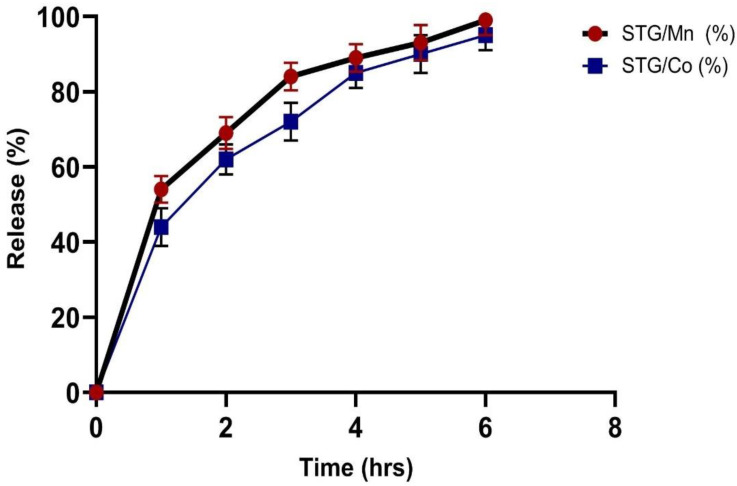
In vitro diffusion profile of STG/Mn and STG/Co complexes in slightly basic phosphate buffer saline at 37 °C. Data are represented as mean ± SD (*n* = 3).

To exploit the use of STG/Mn and STG/Co complexes for an effective drug delivery system, we carried out this experiment (in vitro drug release of) at different time intervals (1, 2, 3, 4, 5, and 6 h), and the resulting data are shown in Figure 8. The in vitro releasing medium was chosen as slightly basic phosphate buffer. The first release was fast within the first 2 h, followed by a gradual release up until 6 h.

### 3.4. Hepatic Enzyme Levels

**Figure 9:**
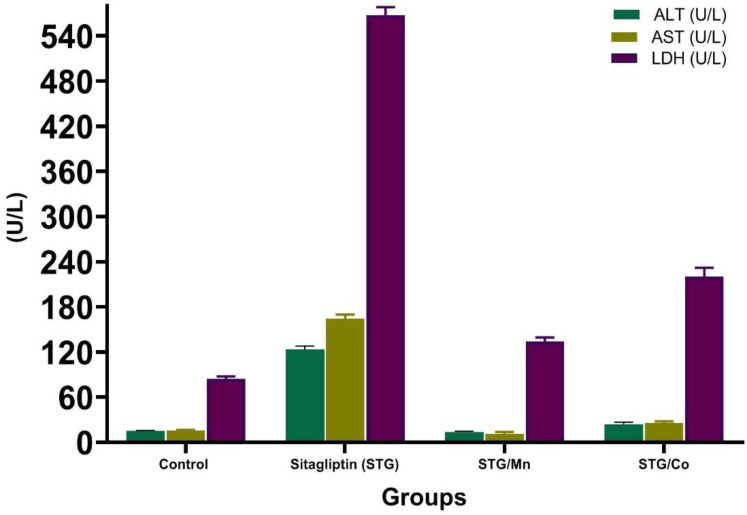
Effects of STG and its metal complexes (STG/Mn and STG/Co) on liver enzyme functions.

ALT, AST, and LDH were significantly elevated in the STG-treated group as compared to the control group (Table 3 and Figure 9). Meanwhile, the treatment of male rats with STG metal complexes either STG/Mn or STG/Co improved all the above parameters better than each one alone, and this returned to the functions of either Co or Mn; Mn is an essential human dietary element. It is involved in several biological processes, metabolism, and free radical defense systems; based on the obtained findings, Mn alters STG chemical structure and forms a new structural compound (STG/Mn) complex after the coordination of Mn with STG via carbonyl and amino groups, and this new formula greatly improved the hypoglycemic and hepatoprotective activities of STG.

Additionally, Co as an essential metal, which is involved in the metabolism of all animals and thus interacts with liver enzymes, and due to the role of bacteria in the stomachs of ruminant animals to convert cobalt salts into vitamin B12, Co therefore markedly improves the health. We proved this in the current finding by obtaining a new structure of STG with Co complex thus giving more characterizations to the efficacy of STG drug.

### 3.5. Oxidative Stress Enzymatic and Nonenzymatic Biomarkers

Table 4 shows the changes in antioxidant enzyme markers in the hepatic tissues of male rats treated with STG. MDA levels of male rats are significantly elevated with all the antioxidant enzymes (SOD, CAT, GRx, and GPx) decreasing. The combination of STG with Mn and Co significantly decreased the MDA levels and improved all the antioxidant enzymes more than treatment with STG alone.

By contrast, the MDA levels in STG/Mn and STG/Co groups significantly declined compared with those in the STG-treated group. Therefore, novel complexes of STG/Mn and STG/Co improved the antioxidant defense system in the hepatic tissue homogenates (Table 4).

These biological data of these novel complexes with STG, either Mn or Co, enhanced the antioxidant capacities of the original ligand as the novel formula ameliorated the hepatic antioxidant activities. So, the use of STG in combination with vital metals such as Mn and Co was attempted to increase the antioxidant effectiveness of STG as it is know that enzymes such as glutathione and superoxide dismutase combine effectively with metals and thus increase their abilities in the scavenging of free radicals. This was clearly apparent in the high scavenging activity of STG with either Mn or Co and the reducing of the lipid peroxidation final marker “MDA” and improving the antioxidant enzymes (SOD, CAT, and GPx).

### 3.6. Histological Examination

**Figure 10:**
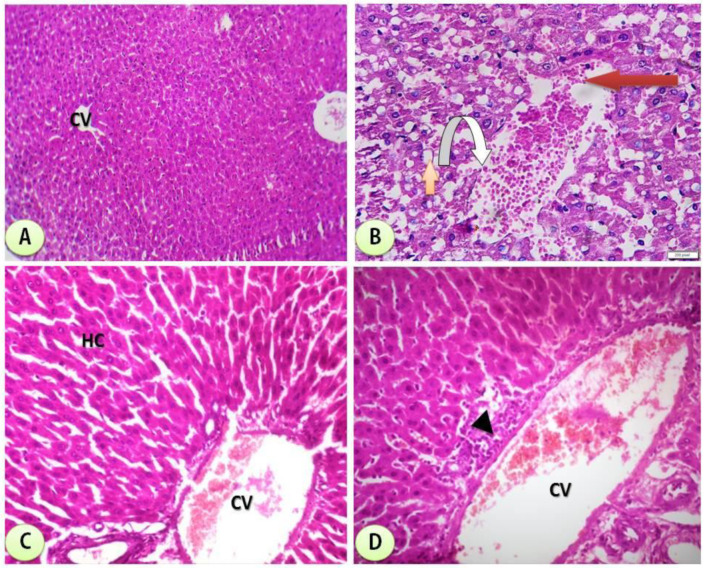
(**A**) Control group: photomicrograph of cross-section of the hepatic tissues showing normal hepatic structure (H&Ex200). (**B**) STG group showing cross-section of experimental rat liver showing some adverse effects of STG administration on the liver comprising loss of normal architecture of liver lobules, ballooning degeneration of hepatocytes with cytoplasmic clearing (steatohepatitis) (orange arrow) and severe dilatation of the central vein with marked hemorrhage within it (red arrow) communicating with some dilated congested blood sinusoids (inverted arrow) (H&EX400). (**C**) STG/Mn-treated group showing amelioration of hepatic tissues with mildly dilated central vein (CV) at the center of the lobule surrounded by normal hepatocytes (HC) (H&Ex200). (**D**) STG/Co-treated group showing almost normal hepatic structure with dilated central vein (CV) at the center of the lobule surrounded by the hepatocytes; pericentral zone hepatocytes shows some necrosis (arrows) with lymphocytic inflammatory cell infiltrate (arrow head) H&Ex200).

Figure 10 is a photomicrograph of hepatic tissues showing normal hepatic structure in the control group. It also shows the hepatic structure alterations with degeneration of hepatocytes and severe dilation of the central vein with marked hemorrhage and clear improvement in groups treated with combination of STG/Mn and STG/Co-treated groups. The scoring of the different treated groups is shown in Table 5.

The structure of hepatic tissues was greatly improved in groups treated with new complexes between STG and Mn and Co, and this proved their capacities in improving biochemical functions of treated groups with STG/Mn and STG/Co and also on the structural levels as clear in the histological sections of different treated groups and the scoring of different fields, and this is an important issue in proving the capacities of the novel complexes on the cellular levels.

### 3.7. Antibacterial Activity Evaluation

**Figure 11:**
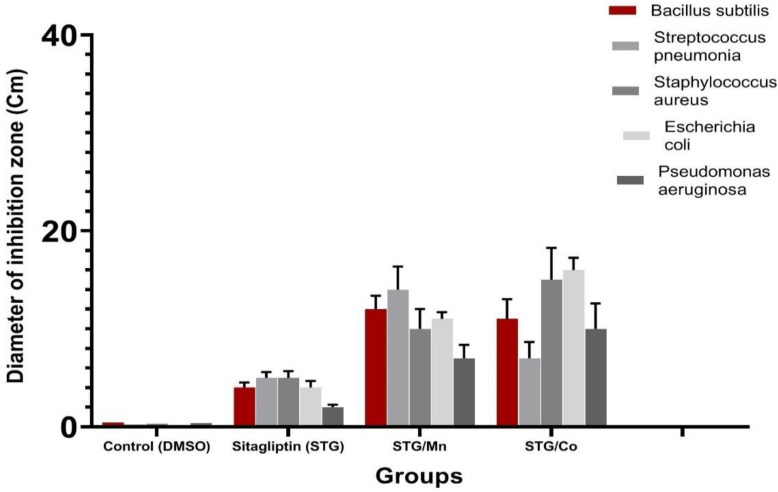
Antibacterial activity of STG and Mn(II) and Co(II) metal complexes.

Biological evaluations were performed of the target complexes against Gram-positive (*Bacillus subtilis*, *Streptococcus pneumonia* and *Staphylococcus aureus*) and Gram-negative (*Escherichia coli* and *Pseudomonas aeruginosa*) bacteria. Results from the agar disc diffusion tests for the antimicrobial activities of the target compounds are presented in Table 6 and illustrated in Figure 11. The diameters of the zone of inhibition (in mm) of the standard drug Amikacin against Gram-positive bacteria *B. subtilis* and *S. aureus* and Gram-negative bacteria *E. coli* and *P. aeruginosa* were found to be 38, 32, 33, and 37 mm, respectively. Under identical conditions, Table 6 and Figure 11 show that all complexes were found to be efficient, with high antimicrobial activity.

## 4. Discussion

It is known that drugs generally affect liver vitality, therefore, previous literature on the subject has confirmed that STG has some adverse effects on hepatic structure and metabolism [1]. Drugs often cause unrecognized and sometimes acute liver injury [35].

STG resulted in a slight decline in both (Alanine transaminase and Aspartate transaminase) AST and ALT in clinical trials as confirmed in such drugs and xenobiotics in previous studies [36]. These results are consistent with the obtained data, as STG-treated groups elicited a significant increase in AST, ALT, and LDH when compared to the normal control group. However, these parameters were greatly improved in groups treated with STG/Mn and STG/Co complexes.

In the current study, metal complexes (Mn and Co) combined with STG resulted in significant improvements in all the aforementioned parameters as compared to the normal control group.

The results presented herein provide compelling evidence regarding the biological effects of STG on liver functions, and the novel effect of STG metal complexes on ameliorating hepatic structure in male rats. First, there were great biochemical and histological differences between the treated rats that received the STG drug alone and those that received STG metal complexes, either Co or Mn.

Manganese (Mn) is an essential human dietary element. It is present as a coenzyme in several biological processes, which include macronutrient metabolism and free radical defense systems. Manganese (Mn) is an essential element that is involved in the synthesis and activation of many enzymes and in the regulation of the metabolism of glucose and lipids in humans [37,38]. The classes of enzymes that have Mn cofactors include oxidoreductases and hydrolases. Other enzymes containing Mn are Mn-containing superoxide dismutase (MnSOD). Mn deficiency in humans results in a number of medical problems.

Cobalt is essential to the metabolism of all animals. It is a key constituent of cobalamin, also known as vitamin B12, the primary biological reservoir of cobalt as an ultratrace element [39] Bacteria in the stomachs of ruminant animals convert cobalt salts into vitamin B12, a compound which can only be produced by bacteria or archaea. A minimal percent of cobalt therefore markedly improves the health, and an uptake of 0.20 mg/kg a day is recommended because they have no other source of vitamin B12 [40].

The results of these studies clearly indicate that the ligands and their complexes are all potent and biologically active against one or more testing bacterial strains. So, the complexing of STG with either Mn and Co will be of great interest and may play a vital role in improving glucose metabolism [41,42,43] as Mn and Co change the chemical structure of STG, which plays a role in the improvement of biological functions, hepatic functions, and enzymes; enhances antioxidant capacities, and elevates SOD enzymes [44,45]. We confirmed this in the current study, and there was great improvement in the groups treated with either Mn or Co metal ions that improved hepatic function enzymes and hepatic histological structures.

We believe that the improvement shown in the animal group treated with either Mn/STG or Co/STG was due to the importance of these metals as an essential element for healthy development. This concept is consistent with a previous study by El-Megharbel et al. [5].

More evidence for the beneficial impact of Mn/STG and Co/STG complexes is that they improve hepatic functions and ameliorate hepatic antioxidant activities. SOD deficiency is believed to be associated with the development of many inflammatory processes [43], and these complexes may perform the role of SOD in detoxification in normal tissues. Therefore, the STG/Mn and STG/Co novel metal complexes greatly improve SOD enzyme levels, indicating high potency for the hepatic antioxidant cellular defense system, as confirmed in a previous study [5].

The use of the SOD enzyme as a therapeutic agent has been partially successful in animals, but not in humans [45]. Pharmacokinetic problems, such as the drug’s quick delivery problems and the drug’s short half-life in the blood, are essential obstacles to the use of antioxidant enzymes in humans. As a result, the use of STG in combination with vital metals such as Mn and Co was attempted to increase the effectiveness of STG while minimizing its hepatic adverse effects. Mimicking natural enzymes such as SOD may have similar chemistry and thus be useful in treating diseases under low toxicity and safe biological conditions.

The main purpose of STG is to lower or maintain blood glucose levels as low as possible, reducing the risk of complications. Owing to the multifactorial nature of the pathophysiology of type II diabetes mellitus, the response to these drugs is individualized and can show a great difference [46].

The cascade of complications involved in inflammatory procedures is due to oxidative stress. The current findings support it and are supported by the current finding that STG novel metal complexes are effective in preventing hepatic toxicity and reducing oxidative injury [47]. Some of these mechanisms are potentially modifiable by dipeptedyl peptidase-4 inhibition [48].

Oral administration of STG and STG metal complexes (Cu, Mg, or Zn) at the indicated dose significantly reduced blood glucose levels during the treatment period and improved glycemic control in diabetic rats, as evidenced by an amelioration in insulin hormone levels [5]. However, the STG metal complexes showed better blood glucose control than either agent alone. Furthermore, an antidipeptedyl peptidase-4 activity could be obtained when combined with STG. This notion could explain the synergistic effect of STG on glucose homeostasis when combined with metals.

The Gram-negative strains are exposed to various stress conditions during pathogenesis, of which acid stress serves as a major defense mechanism in the host [49]. Such environments are encountered, and this supports the main hypothesis of the study that proved the hepatoprotective and antioxidant capacities of two synthesized STG with either Co(II) or Mn(II). The two complexes also showed antibacterial activity in addition to their abilities in reducing free radical production.

## 5. Conclusions

This study revealed that hepatic tissues were greatly ameliorated after administration of STG complexes with Mn(II)/STG and Co(II)/STG combination therapy, which improved liver enzymes and contributed to improvements in hepatic and metabolic complications by a different percentage over STG alone. Our results confirm that the Mn/STG and Co/STG complexes were efficient and safe for the treatment of hepatic alterations and oxidative stress with increased antioxidant activity, also improving the histological structure of the liver tissues. The STG/Mn complex was highly effective against *Bacillus subtilis* and *Streptococcus pneumonia,* while STG/Co was highly effective against *Escherichia coli, Pseudomonas aeruginosa*, and *Staphylococcus aureas*. These results open a new door for developing novel therapeutic strategies for improving health, and we recommend more prospective clinical studies on the safety of Mn/STG and Co/STG complexes and a wide range of other metals in a wide range setting to gain a better understanding of their effectiveness in protecting hepatic tissues.

## Figures and Tables

**Table 1:** Instrumentations (All Analyses Were Performed after Preparation of the Complexes Immediately).

Instrument	Measurement
Perkin Elmer CHN 2400 (USA)	The contents C, H, and N
Jenway 4010 conductivity meter	Electrolytic or nonelectrolytic character
Bruker FTIR Spectrophotometer (4000–400 cm^−1^)	IR measurements
Quanta FEG 250 equipment	Scanning electron microscopy (SEM) images
X ‘Pert PRO PAN analytical X-ray powder diffraction, target copper with secondary monochromate	The X-ray diffraction patterns
JEOL 100 s microscopy	The transmission electron microscopy images (TEM)
Flame atomic absorption spectroscopy instrument (Perkin Elmer Analyst 400)	Metal content and Cl

**Table 2:** Assignments of infrared spectral bands for STG and its complexity.

Assignments	Compounds		
	STG	Mn(II)	Co(II)
N–H stretching	3357	3300	3331
C–H stretching	3059 2917 2850	2920 2856	2917 2849
C=O stretching	1669	1637	1638
C=N stretching	1633	1633	1633
NH_2_ bending	1609	-	-
C–C and C–N stretching	1556 1514 1426	1518	1517 1428
CH in plane bending	1324 1274	1334 1316 1275	1333 1276
C–F stretching	1330 1267 1146	1331 1269 1140	1332 1270 1139
CH out-of-plane bending	915 880 841 720	887 840 663	887
M–O stretching	-	660	666
M–N stretching	-	560	558

**Table 3:** Effects of STG, STG/Mn, and STG/Co on liver function levels in male rats.

Parameters	Control	STG	STG/Mn	STG/Co
ALT (U/L)	12.51 ± 0.42 ^c^ (−−−)	123.31 ± 6.69 ^a^ (++++)	13.40 ± 0.82 ^bc^ (−−−)	23.87 ± 3.14 ^b^ (−−−)
AST (U/L)	12.43 ± 0.37 ^c^ (−−−)	164.26 ± 5.27 ^a^ (++++)	11.27 ± 0.52 ^c^ (−−−)	25.53 ± 3.20 ^b^ (−−−)
LDH (U/L)	84.05 ± 8.47 ^c^ (−−−)	567.42 ± 47.00 ^a^ (++++)	133.56 ± 11.91 ^c^ (−−−)	220.02 ± 12.05 ^b^ (++−)

Results are expressed as mean ± SE. Symbols are different alphabetically to indicate a significant comparison compared to the control group and other treated groups (*p* < 0.05) (Similar letters imply partial or complete nonsignificance). STG, Sitagliptin; ALT, Alanine aminotransferase; AST, Aspartate aminotransferase; LDH, Lactate dehydrogenase. Scoring of hepatic enzymes: (++++), moderate hepatic damage; (++−), slight hepatic damage; and (−−−), no hepatic damage.

**Table 4:** Effects of STG, STG/Mn, and STG/Co on oxidative stress enzymes and oxidative damage markers in hepatic tissues in male rats.

Parameters	Control	STG	STG/Mn	STG/Co
CAT (U/g)	7.68 ± 0.26 ^b^	1.79 ± 0.16 ^c^	7.00 ± 1.11 ^a^	6.87 ± 0.35 ^b^
SOD (U/g)	8.19 ± 0.26 ^b^	2.07 ± 0.45 ^d^	7.83 ± 0.34 ^a^	7.86 ± 0.39 ^c^
GRx (U/g)	6.46 ± 0.38 ^a^	1.30 ± 0.88 ^c^	6.12 ± 0.33 ^a^	6.09 ± 0.45 ^b^
MDA (µg/mg)	2.75 ± 0.15 ^c^	55.21 ± 1.30 ^a^	5.71 ± 0.68 ^b^	4.64 ± 0.68 ^b^
GPx (U/g)	12.22 ± 0.45 ^a^	2.55 ± 0.40 ^c^	11.72 ± 0.51 ^a^	11.56 ± 0.65 ^b^

Results are expressed as mean ± SE. Symbols are different alphabetically to indicate a significant comparison compared to the control group and other treated groups (*p* < 0.05) (Similar letters imply partial or complete nonsignificance). Similar letters imply partial or complete nonsignificance. STG, Sitagliptin; CAT, Catalase; MDA, Malondialdehyde; SOD, Super oxide dismutase; GRx, Glutathione reductase; and GPx, Glutathione peroxidase.

**Table 5:** Scoring of histological hepatic structures.

Scoring		Control Group	STG Group	STG/Mn	STG/Co
	Groups				
Normal hepatic structure		(++++)	(++−)	(+++)	(+++)
Loss of normal hepatic lobules		(−−−−)	(++++)	(−−−−)	(−−−−)
Marked hemorrhage		(−−−−)	(++−)	(−−−−)	(−−−−)
Dilated congested blood sinusoids		(−−−−)	(++++)	(−−−−)	(−−−−)
Mildly dilated central vein		(−−−−)	(−−−−)	(+++)	(++−)
Lymphocytic inflammatory cell infiltration		(−−−−)	(−−−−)	(−−−−)	(+++)

Scoring indicates: (++++), High effect; (+++), Moderate effect; (++−), Slight effect; and (−−−−) No effect.

**Table 6:** Inhibition zone diameter (Cm/mg sample) of STG metal complexes.

Sample	Inhibition Zone Diameter (mm/mg Sample)				
	*Bacillus subtilis* (G^+^)	*Streptococcus* *pneumonia* (G^+^)	*Staphylococcus* *aureas* (G^+^)	*Escherichia coli* (G^−^)	*Pseudomonas* *aeruginosa* (G^−^)
Control (DMSO)	0.0 ± 0.0 ^c^	0.0 ± 0.0 ^d^	0.0 ± 0.0 ^e^	0.0 ± 0.0 ^d^	0.0 ± 0.0 ^d^
Sitagliptin (STG)	4 ± 0.01 ^b^	5 ± 0.53 ^c^	5± 0.39 ^d^	4 ± 0.21 ^c^	2 ± 0.15 ^c^
Mn(II)–STG	12 ± 0.12 ^a^	14 ± 0.31 ^a^	10 ± 0.48 ^b^	11 ± 0.16 ^a^	7 ± 0.25 ^a^
Co(III)–STG	11 ± 0.24 ^a^	7 ± 0.21 ^b^	15 ± 0.37 ^c^	16 ± 0.45 ^a^	10 ± 0.49 ^a^

Means within the same column (mean ± SE) with different letters are significant at *p* ≤ 0.05 using Duncan’s multiple range test, where the highest mean value has symbol (a) and those decreasing in value are assigned alphabetically.

## Data Availability

All the data are available inside the text.
